# Effectiveness and safety of fire needle on periarthritis of shoulder: Protocol for a systematic review and meta-analysis

**DOI:** 10.1097/MD.0000000000015673

**Published:** 2019-05-17

**Authors:** Cihui Huang, Linling Xie, Yunxin Lin, Liang Zheng

**Affiliations:** a Guangzhou University of Chinese Medicine; b Acupuncture and Massage Department, The First Affiliated Hospital of Guangzhou University of Chinese Medicine, Guangzhou, China.

**Keywords:** fire needle, periarthritis of shoulder, protocol, systematic review

## Abstract

**Background:**

Fire needle which is an integral part of the acupuncture therapy. Periarthritis of shoulder (PAS) is a common disease, which brings lots of pain for patients. The clinical practice indicates that fire needle has a therapeutic effect on the PAS. Here, we will provide a protocol to explore the effectiveness and safety of fire needle for the PAS.

**Methods:**

We will search the randomized controlled trails (RCT) literatures of fire needle for the PAS in 9 electronic databases, including 5 English databases (PubMed, Web of Science, EMBASE, the Cochrane Central Register of Controlled Trials [Cochrane Library], and WHO International Clinical Trials Registry Platform [TCTRP]) and 4 Chinese databases (Chinese National Knowledge Infrastructure [CNKI], Chinese VIP Information, Wanfang Database, and Chinese Biomedical Literature Database [CBM]). We will consider the Ability assessment of daily living activities (ADL) as the primary outcome and the secondary outcome will include visual analogue scale (VAS), shoulder range of motion (ROM) and adverse events incidence caused by fire needle, such as dizziness, nausea, vomiting, weariness, etc. The selection of the studies will be performed by EndnoteX7 software. All analyses will be conducted by using RevMan software V5.3.

**Result:**

This study will provide a rational synthesis of current evidences for fire needle on PAS.

**Conclusion:**

The conclusion of this study will provide evidence to judge the effectiveness and safety of fire needle on PAS.

**Registration:**

PROS-PERO CRD42019119686.

## 1 Introduction

Periarthritis of shoulder (PAS), which is named the frozen shoulder, is a common, disabling musculoskeletal disorder in middle-aged people. It commonly refers to a collection of pain symptoms in shoulders, plus motor function limitation, because soft tissue abnormalities surrounding the affected shoulder.^[1]^ The PAS is prevailing in people who are 40 to 60 years, and thus they were called “the forty's shoulder.” The population incidence of PAS is 2% to 5% in the United States.^[2]^ In China, the PAS in urban population accounts for 8% and 45% are female patients, and the PAS can have serious influences on patients’ health and quality of life. Nowadays, there are numerous treatment options for PAS, including intra-articular triamcinolone injection^[3]^ and bupivacaine suprascapular nerve blocks.^[4]^ These modern therapies are successfully employed in clinical practice for PAS treatment, but the treatment effects are not always long-lasting and most of which disappear in 4 weeks after treatment approximately.^[5,6]^ Moreover, these treatment options may cause severe adverse events, such as infectious arthritis and cartilage damage.^[7]^ Therefore, numerous research studies have been carried out on this topic to reduce the symptoms of PAS with efficiency and safety.

In China, acupuncture is an effective traditional therapeutics. During over 3000 years of history, acupuncture treatment gets developed and evolved to treat various disorders. Fire needle is one of the acupuncture therapy which was first recorded in Huangdi Neijing, one of the earliest medical books in China.^[8]^ Taking into account patient tolerance, three filiform needles were tied together. The depth of insertion and the needle spacing were determined based on our own experience.^[9–11]^ Fire needle has the functions of enhancing immunity, regulating blood circulation, and preventing diseases. Some clinical trials have found that fire needle has a significant impact on PAS. In addition, it has the advantages of safe, reliable, and is easy to use without toxic and side effects. However, relationships between fire needle and PAS have not been revealed clearly. Thus, the purpose of this review is to summarize clinical researches on fire needle for PAS and findings of this review will be reliable within evidence of clinical studies. This review only focuses on the effects of fire needle on PAS rather than other effective treatments.

The aim of this study is to systematically review current available literature to assess the efficacy and safety of the fire needle treatment for PAS.

## 2 Methods

### 2.1 Study registration

The protocol has been registered on the International Prospective Register of Systematic Reviews (PROSPERO) (registration number, CRD42019119686) basing on the Preferred Reporting Items for Systematic Reviews and Meta-Analyses Protocols (PRISMA-P) statement guidelines.

### 2.2 Ethics and dissemination

Ethical approval is not required as there are no issues about participants’ privacy in our research. We aim to publish the results in a peer-reviewed journal. The results of this review will provide information about the safety and efficacy of fire needle treatment for PAS and help clinicians make decisions on clinical practice.

### 2.3 Inclusion criteria for study selection

#### 2.3.1 Types of studies

All available randomized controlled trials (RCTs) on fire needle treatment for PAS will be included. Others such as retrospective study, case report, review, and studies which use inappropriate random sequence generation methods will be excluded. Language will be restricted to Chinese and English.

#### 2.3.2 Types of participants

We will include studies on patients that have been diagnosed as PAS by clinicians that refer to the guiding principles for clinical research of new Chinese medicine and standard diagnostic criteria of the second national seminar for periarthritis of the shoulder.^[12]^ There will be no restriction on age, gender, ethnicity, and profession.

#### 2.3.3 Types of interventions

The purpose of the study is on clinical trials of fire needle treatment for PAS. Studies applied fire needle in the experimental group will be included. Fire needle combined with other therapies will be excluded if the efficacy of fire needle cannot be clarified in the combined therapy. The therapeutic intervention of controlled group can be conventional acupuncture, electro-acupuncture, auriculo-acupuncture, or pharmcological therapy.

#### 2.3.4 Types of outcome measures

##### 2.3.4.1 Primary outcome

The primary outcome is the ability assessment of daily living activities (ADL),^[13]^ which is comprised of 8 items.

##### 2.3.4.2 Secondary outcomes

- Visual analogue scale (VAS)^[13]^
- Shoulder range of motion (ROM)^[13]^
- Adverse events incidence caused by fire needle, such as dizziness, nausea, vomiting, weariness, etc

### 2.4 Search methods for study identification

#### 2.4.1 Electronic searches

We will search PubMed, MEDLINE, EMBASE, Cochrane Library, China National Knowledge Infrastructure (CNKI), Wanfang data, Chinese Scientific Journals Database (VIP) and China biomedical literature database (CBM) to acquire eligible studies published up to December 2018. Various combinations of Medical Subject Headings and non-MeSH terms will be used, including “periarthritis of shoulder,” “the frozen shoulder,” “needle,” “acupuncture,” “fire needle,” “Electroacupuncture,” and “huo zhen,” which will be searched individually or in combination. Language, population or country restrictions will not be applied.

The specific search strategy will be (taking PubMed as an example):

#1 periarthritis of shoulder [MeSH] OR the frozen shoulder [Title/Abstract]
#2 acupuncture [Title/Abstract]OR fire needle [Title/Abstract] OR Electroacupuncture

[Title/Abstract] OR huo zhen [Title/Abstract]

#3 #1 AND #2.

We will modify the strategy for other databases searching.

#### 2.4.2 Searching other resources

Relevant systematic review or meta-analysis of RCTs will be electronically searched. Moreover, we will filter relevant medical journals and magazines to identify literature which is not included in the electronic databases.

### 2.5 Data collection and analysis

#### 2.5.1 Study selection

We will select the RCTs comparing the effect and safety of fire needle therapy on PAS. Those articles meeting one of following items will be excluded:

1. the duplicates,

2. the participants did not meet the diagnosis criteria of PAS or the diagnosis criteria is unknown,

3. not RCT studies,

4. the studies in which the experimental participants don’t receive fire needle therapy in combination with conventional therapy as the primary intervention,

5. the intervention contains any other traditional Chinese medicine (TCM) therapy, and

6. incomplete data which will be needed.

**Figure 1:**
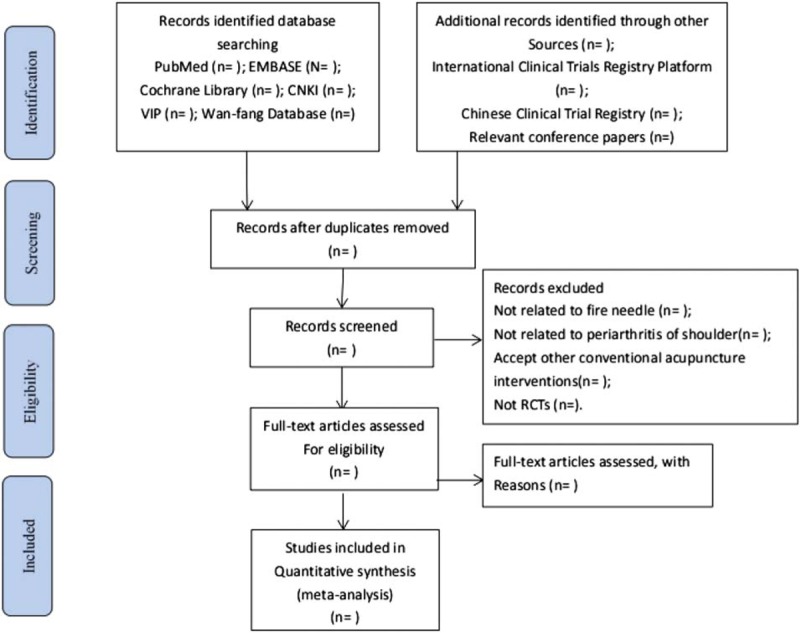
Flow diagram of study selection process.

The studies whether are eligible will be assessed by two authors. When there is any disagreement during the articles inclusion, we will discuss to solve it. The specific process of studies screening will be displayed in a Preferred Reporting Items for Systematic Reviews and Meta-Analyses (PRISMA) flow diagram (Fig. 1).

#### 2.5.2 Data extraction and management

Two authors will extract the date independently. We will discuss any divergence raised during the data extraction process. The following information will be extracted: study details (authors, country, year of publication, multicenter or single center study), participant details (baseline data, diagnostic criteria), the methods used (registry platform, sample size, blinding method), the interventions and the outcomes (ADL score, VAS score, ROM, and adverse events incidence). We will contact the corresponding author for the unreported data.

#### 2.5.3 Assessment of risk of bias in included studies

According to the *Cochrane Handbook*, the risk of bias of each pooled study will be independently evaluated for Systematic Reviews of Interventions.^[14]^ Following seven aspects will be evaluated and described for the risk judgment rationally including random sequence generation, allocation concealment, blinding of participants and personnel, blinding of outcome assessments, incomplete outcome data, selective reporting, and other bias. The risks will be categorized as three levels: “low risk of bias,” “high risk of bias,” and “unclear risk of bias.”

#### 2.5.4 Measures of treatment effect

For continuous variables, we will use mean difference to evaluate the extracted data. For dichotomous variables, rate ratio (RR) will be applied to analyze. The confidence intervals (CIs) for both continuous and dichotomous variables will be set to 95%.

#### 2.5.5 Dealing with missing data

The corresponding author will be contacted by telephone or e-mail for insufficient or missing trial data. If the missing data cannot be supplied or failure to contact the author, a limited analysis will be performed which based on the available data and we will discuss the potential impact of the missing data.

#### 2.5.6 Assessment of heterogeneity

The heterogeneity will be evaluated with the use of *I*^2^ values in accordance with the *Cochrane Handbook* (0–40%, might not be important, 30–60%, may represent moderate heterogeneity, 50–90%, may represent substantial heterogeneity, and 75–100% may represent considerable heterogeneity). We will select the random effects model and then further subgroup analysis will be performed to investigate the possible causes of heterogeneity, if the heterogeneity among trials is significant (*I*^2^ ≥ 50%). Conversely, we will choose the fixed effect model, if an *I*^2^ values less than 50%.

#### 2.5.7 Assessment of reporting bias

Funnel plot will be used to assess reporting biases of the studies include. We will consider that the reporting bias is existing and the reliability is low if the points on both sides of the funnel plot are dispersed and asymmetrical. Conversely, if the points on either side of the funnel plot are symmetrically distributed in substantial, we will consider the reporting bias as non-existent and the result is reliable.

#### 2.5.8 Data synthesis and subgroup analysis

We will use RevMan software (V5.3, The Nordic Cochrane Centre, The Cochrane Collaboration, Copenhagen, Denmark) to conducted all analyses. And we will select a random effects model or fixed effects model to merge the primary and secondary outcome indicators in accordance with the results of heterogeneity test. We will apply the fixed effects model for data synthesis of low heterogeneity (*I*^2^ < 50%) while the random effects model will be conducted if the heterogeneity is significant (*I*^2^ ≥ 50%). It is considered that differences are statistically significant if the results of *Z* test show that *P* value is less than .05, and the 95% CI dose not contain 0 (for continuous variables) or the 95% CI dose not contain 1 (for dichotomous variables).

If heterogeneity is evaluated as significant (*I*^2^ ≥ 50%) and the trials included are adequate, we will perform a subgroup analysis to explore the potential source of the heterogeneity according to the difference in participant characteristics, interventions, controls, and outcome measures.

#### 2.5.9 Sensitivity analysis

We will carried out sensitivity analysis to identify the quality and robustness of the meta-analysis result when the outcome analyses involve a large degree of heterogeneity, according to sample size, methodological quality, and the effect of missing data.

#### 2.5.10 Grading the quality of evidence

We will evaluate the quality of evidence and rate it into four levels: high, moderate, low, or very low in accordance with the Recommendations Assessment, Development and Evaluation (GRADE) guidelines.^[15]^

#### 2.5.11 Ethics and dissemination

Ethical approval will not be necessary because the data included in our study are derived from published literature and are not linked to individual patient data. The systematic review providing implication of the effectiveness and safety of fire needle for PAS will be published in a peer-reviewed journal or conference presentations.

## 3 Discussion

The PAS which commonly pathologically encompasses aseptic inflammation and soft tissue adherence in shoulder joint, is a prevailing musculoskeletal disorder. The PAS seriously causes pain, leads to notable motor function limitation of the affected joint, and subsequently endangers people's life quality, though not a fetal disorder or major disease like cancer, angina pectoris, stroke, and so forth. Thus, the treatment of PAS is of great significance to increase people's life quality and advance public health.^[16]^ However, conventional treatment of modern medicine that are associated with adverse events and side effect, cannot withdraw the symptoms and harmfulness of PAS completely. Therefore, it is necessary to search for alternative therapy with higher effective rate and less side effects.

Fire needle is an extensively accepted alternative therapy, as an important part of complementary and complementary medicine, has gained increased popularity for the management of constipation around the world.^[17]^ Fire needle can relieve pain, improve the blood circulation, stimulate metabolism of local tissue, etc.^[18]^

However, the evidence of efficacy and safety of fire needle is insufficient and the underlying mechanisms remain largely unknown. Therefore, it is imperative to perform a systematic review and meta-analysis of available literature to evaluate the clinical efficacy and safety of fire needle on PAS objectively.

To the best of our knowledge, it will be the first systematic review and meta-analysis on fire needle in the treatment of PAS. First, the results of this review will provide objective statistics for further researches on fire needle. Secondly, the results will offer reliable references for clinicians and patients in the treatment of PAS with fire needle. Thirdly, the results may introduce an alternative therapy of PAS to policy makers to decrease the burden of public health.

## Author contributions

Cihui Huang, Linling Xie, and Liang Zheng designed the study. Cihui Huang and Yunxin Lin drafted the protocol. All authors revised the manuscript. All authors approved the final version.

**Conceptualization:** Cihui Huang, Linling Xie, Yunxin Lin.

**Methodology:** Cihui Huang, Liang Zheng.

**Supervision:** Liang Zheng.

**Writing – original draft:** Cihui Huang, Linling Xie.

**Writing – review & editing:** Cihui Huang, Linling Xie, Yunxin Lin.
